# Genome-wide association study identifies COL2A1 locus involved in the hand development failure of Kashin-Beck disease

**DOI:** 10.1038/srep40020

**Published:** 2017-01-06

**Authors:** Jingcan Hao, Wenyu Wang, Yan Wen, Xiao Xiao, Awen He, Cuiyan Wu, Sen Wang, Xiong Guo, Feng Zhang

**Affiliations:** 1 Key Laboratory of Traece Elements and Endemic Diseases of National Health and Family Planning Commission, School of Public Health, Health Science Center, Xi’an Jiaotong University, Xi’an, P. R. China

## Abstract

Kashin-Beck disease (KBD) is a chronic osteochondropathy. The pathogenesis of growth and development failure of hand of KBD remains elusive now. In this study, we conducted a two-stage genome-wide association study (GWAS) of palmar length-width ratio (LWR) of KBD, totally including 493 study subjects. Affymetrix Genome Wide Human SNP Array 6.0 was applied for genome-wide SNP genotyping of 90 KBD patients. Association analysis was conducted by PLINK. Imputation analysis was performed by IMPUTE against the reference panel of the 1000 genome project. Two SNPs were selected for replication in an independent validation sample of 403 KBD patients. In the discovery GWAS, significant association was observed between palmar LWR and rs2071358 of COL2A1 gene (*P* value = 4.68 × 10^−8^). In addition, GWAS detected suggestive association signal at rs4760608 of COL2A1 gene (*P* value = 1.76 × 10^−4^). Imputation analysis of COL2A1 further identified 2 SNPs with association evidence for palmar LWR. Replication study observed significant association signals at both rs2071358 (*P* value = 0.017) and rs4760608 (*P* value = 0.002) of COL2A1 gene. Based on previous and our study results, we suggest that COL2A1 was a likely susceptibility gene involved in the hand development failure of KBD.

Kashin-Beck disease (KBD) is a chronic, endemic osteochondrosis^1^,^2^. KBD is usually first occurred in children aged 3–12 years. With age, KBD patients gradually exhibit finger flexion, enlarged finger joints, shortened fingers and other features caused by impaired epiphyseal growth and ossification^3^. About 30 million people living in KBD prevalent areas are at the risk of KBD in China^4^. Based on severity of joint lesions, KBD is clinically classified into three grades. Comparing with grade I KBD patients, grade II and III KBD patients have significant skeletal growth and development failure, such as shortened fingers, shortened humeri and short stature.

The molecular mechanism of KBD remains elusive now. Recently, a study using 10,823 subjects from 1,361 families observed significantly familial clustering of KBD patients^5^. The estimated heritability of KBD achieved 37.20% in families^5^. Several susceptibility genes have been identified for KBD, including GPX1^6^,^7^, HLA-DRB1^8^, ABI3BP^9^, GPX4^10^, COL9A1^11^, SEPS1^12^, ITPR2^13^, CD2AP^14^ and TNF^19^. However, the genetic risk of KBD explained by reported susceptibility genes are limited, suggesting the existence of additional susceptibility genes. Hands are the major skeletal sites affected by KBD. Because enlarged finger joints are the representative clinical manifestations of KBD, almost all of previous KBD studies focused on finger joint deformities. They used enlarged finger joints as study phenotypes and treated KBD as a qualitative trait. To the best of our knowledge, no genetic study of the growth and development failure of hand of KBD has been conducted by now. This may miss the susceptibility genes involved in the skeletal development failure of KBD.

In this study, we conducted a two-stage genome-wide association study (GWAS) of KBD using palmar length-width ratio (LWR) of hands as study phenotype. In the discovery stage, a GWAS of palmar LWR was conducted using 90 representative grade II or III KBD patients. In the replication stage, the significant association signals detected in the GWAS were further validated using an independent sample of 403 KBD patients.

## Results

### Basic characteristics of study samples

In the GWAS, the 90 study subjects contained 50 males and 40 females. The average values and standard deviations (denote as mean ± standard deviation) of palmar LWR, age, height and weight of the 90 study subjects were 0.55 ± 0.08, 59.14 ± 8.40 (years), 148.82 ± 15.70 (cm) and 47.01 ± 8.99 (kg), respectively (Table 1). In the replication study, the 403 study subjects consisted of 169 males and 234 females. The average values of palmar LWR, age, height and weight of the 403 subjects were 0.49 ± 0.04, 59.25 ± 8.82, 154.26 ± 10.44 and 52.11 ± 10.40, respectively (Table 1).

### GWAS and imputation analysis

**Figure 1:**
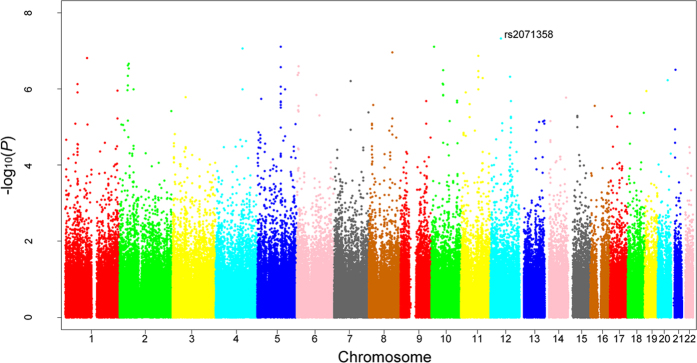
Manhattan plot of GWAS results. X-axis shows chromosomal positions. Y-axis shows –log_10_ (*P* values).

All GWAS subjects were clustered together as one homogeneous sample in the STRUCTURE analysis. EIGENSTRAT analysis calculated a genomic control inflation factor λ = 1.07. After quality control filtering, a total of 532,894 SNP were used for GWAS of palmar LWR of KBD (Fig. 1). Across the whole genome, one significant association signal was observed at the rs2071358 of COL2A1 gene (*P* value = 4.68 × 10^−8^). In addition, GWAS detected suggestive association signal at the rs4760608 of COL2A1 gene (*P* value = 1.76 × 10^−4^). Further imputation analysis identified 2 SNPs, including rs3782915 (*P* value = 4.22 × 10^−7^) and rs740024 (*P* value = 9.34 × 10^−5^). The basic information of the identified SNPs was summarized in Table 2.

### Replication study

To validate the association between COL2A1 and palmar LWR, two genotyped SNP rs2071358 and rs4760608 identified by the GWAS, were selected for replication study. Replication association analysis observed that rs2071358 (*P* value = 0.017) and rs4760608 (*P* value = 0.002) of COL2A1 gene were also significantly associated with the palmar LWR of KBD at Bonferroni corrected significance threshold *P* value < 0.025.

## Discussion

Besides finger joint deformities, the growth and development failure of hand is another important outcome of KBD. To the best of our knowledge, no genetic study of palmar LWR of KBD has been conducted by now. To identify the genes involved in the growth and development failure of KBD, we conducted a two-stage GWAS of palmar LWR of KBD. Our results suggest that COL2A1 gene might be involved in the hand development failure of KBD.

COL2A1 gene encodes the alpha-1 chain of type II collagen, which is specific for cartilaginous tissues. Type II collagen have four isoforms, including IIA, IIB, IIC and IID^16^. They are expressed in a developmentally regulated manner in chondrogenic tissue. For instance, the IIA isoform is mainly expressed in chondroprogenitor cells, while IIB mRNA is predominantly expressed in differentiated chondrocytes^17^,^18^,^19^. Type II collagen is the major extracellular matrix (ECM) component, which makes up about 50% of all protein in cartilage.

It is well documented that type II collagen is essential for the normal embryonic development of the skeleton and linear growth. For instance, a recent study demonstrated that type II collagen was required for proper long bone development of mice^20^. Collagen II knock-out mice didn’t form epiphyseal growth plate^21^. In human, COL2A1 mutation is capable of causing bone dysplasia, characterized by skeletal dysplasia, short stature, and sensorial defects^22^,^23^.

KBD patients have obvious skeletal growth retardation, such as shortened fingers, shortened humeri and short statue. Previous studies have observed that dysfunction of type II collagen contributed to the cartilage lesion of KBD patients. For instance, the expression level of type II collagen in KBD articular cartilage was significantly lower than that in healthy articular cartilage^24^,^25^. Serum type II collagen can serve as the biomarker of the joint damage of KBD^26^. However, no genetic evidence supporting the role of type II collagen in the hand development failure of KBD has been reported by now. Based on previous functional study results of type II collagen and this study results, we infer that COL2A1 was likely implicated in the hand development failure of KBD.

To reveal the functional significanceof the SNPs identified by this study, we aligned the identified SNPs with public expression quantitative trait loci (eQTLs) and methylation quantitative trait loci (meQTLs) database. We did not find overlapped SNP between eQTLs/meQTLs lists and the identified SNPs. We also aligned the identified SNPs withpublished GWAS results of height. No significant association between height and the identified SNPs has been reported by now. This may be partly explained by that the identified SNPswereclose to the causal genetic variations, which were not detected by this study. Additionally, only the genotyped SNP rs2071358 and rs4760608 identified by the discovery GWAS, were selected for replication study. Because rs2071358 and rs4760608 are linkage disequilibrium with other identified SNPs, we believe that the association signals of rs2071358 and rs4760608 in the replication study generally represented the association between COL2A1 and KBD LWR. Further fine mapping and biological studies are needed toconfirm our finding and clarify the potential mechanism of the identified SNPs involved in the hand development failure of KBD.

In summary, we conducted the first GWAS of palmar LWR of KBD. Our study results suggest that COL2A1 was a likely susceptibility gene of KBD. COL2A1 may be implicated in the growth and development failure of hand of KBD. This study may provide new clues for clarifying the pathogenesis and rationale of therapies for KBD.

## Materials and Methods

### Human subjects

In this two-stage GWAS, we totally used four hundred and ninety three Chinese Han subjects, which were previously recruited for genetic studies of KBD^13^. In the discovery study, we used 90 representative grade II or III KBD patients^13^. The 90 KBD patients were collected from Linyou county of Xi’an city of Shaanxi Province in China^13^. In the replication study, another independent sample of 403 KBD patients having LWR data, was recruited from Yongshou county and Bin county of Xi’an city of Shaanxi Province in China^13^. All subjects underwent careful clinical examination and radiography of hands. Palmar length and width of left hand were measured using a digital caliper with a resolution of 0.01 mm. Clinical data of each participant was recorded by nurse-administered questionnaire, including self-reported ethnicity, lifestyle characteristics, health status, family and medical histories. According to the KBD Chinese diagnosis criteria (WS/T207-2010), KBD was diagnosed by at least two experts specialized in KBD. We excluded the subjects with genetic bone and cartilage diseases, primary osteoarthritis and rheumatoid arthritis. More informationabout study subjects can be found in our previous study^13^. 5 mL peripheral blood was drawn from each participant.

### Genotyping and quality control

Genomic DNA specimens were extracted from peripheral blood leukocytes using the E.Z.N. A Blood DNA Midi Kit (Omega Bio-tek, Norcross, USA) following manufacturers’ protocol. Affymetrix Genome Wide Human SNP Array 6.0 (Affymetrix, Santa Clara, CA, USA) was applied for genotyping. Fluorescence intensities were quantified by Affymetrix 30007 G scanner (Affymetrix). To control the impact of cryptic relatedness on our study results, all GWAS samples passed a pairwise identity by descent testing-basedgenetic relatedness check implemented by PLINK^1^,^3^. No genetically related sample (pi-hat > 0.2) was detected in this study. For quality control, the SNPs with Hardy-Weinberg Equilibrium testing *P* values < 0.001, call rates < 98.00% or minor allele frequencies (MAF) < 0.05 were excluded.

### Association study

The genetic background of GWAS samples was evaluated by STRUCTURE software^27^. The genomic control inflation factor λ was calculated by ELGENSTRAT software^28^. With PLINK software, a logistic regression model assuming additive genetic effects was used for association analysis adjusting for sex as a covariate^29^. Significant associations were identified at genomic control corrected *P* value < 5.0 × 10^−8^.

### Imputation analysis

Ungenotyped SNPs in COL2A1 gene were imputed by IMPUTE 2.0 (http://mathgen.stats.ox.ac.uk/impute/impute.html) against the Chinese reference panel of the 1000 genome project (Phase I integrate data version 3)^30^. For quality control, the imputed SNPs missing rates > 0.02, MAF < 0.05 or HWE testing *P* values < 0.001 were excluded.

### Replication study

Based on the Chinese SNP data of the Hapmap project (http://hapmap.ncbi.nlm.nih.gov/), two SNPs rs2071358 and rs4760608 of COL2A1 gene were selected for replication in the independent validation sample of 403 KBD patients. Sequenom MassARRAY platform (Sequenom, San Diego, CA, USA) was applied for genotyping according to the manufacturer’s protocol. The MassARRAY Assay Design 3.1 (Sequenom, San Diego, CA, USA) was used to design the primers of polymerase chain reaction experiment. Sequenom MassArray TYPER 4.0 (Sequenom, San Diego, CA, USA) was used for SNP calling. Association analysis was conducted by PLINK software adjusting for sex as a covariate. Significant associations were identified at Bonferroni corrected significance threshold *P* value < 0.025.

### Ethics Statement

This study was approved by the Institutional Review Board of Xi’an Jiaotong University (Project Number 2014008). The study was conducted in accordance with the principles of Helsinki Declaration. Written Informed consent was obtained from all subjects.

## Additional Information

**How to cite this article:** Hao, J. *et al*. Genome-wide association study identifies COL2A1 locus involved in the hand development failure of Kashin-Beck disease. *Sci. Rep.* **7**, 40020; doi: 10.1038/srep40020 (2017).

**Publisher's note:** Springer Nature remains neutral with regard to jurisdictional claims in published maps and institutional affiliations.

## Figures and Tables

**Table 1:** Basic characteristics of study subjects.

	GWAS^a^	Replication study
Number	90	403
Male/female	50/40	169/234
Average LWR^b^	0.55 ± 0.08	0.49 ± 0.04
Average age (year)	59.14 ± 8.40	59.25 ± 8.82
Average height (cm)	148.82 ± 15.70	154.26 ± 10.44
Average weight (kg)	47.01 ± 8.99	52.11 ± 10.40

^a^GWAS, genome-wide association study.

^b^LWR, length-width ratio.

**Table 2:** Association analysis results of COL2A1 in the discovery GWAS.

SNP	Type	Allele	MAF	D’	r^2^	β (SE)	*P* value
rs2071358	genotyped	A/C	0.356	—	—	−0.501 (0.099)	4.68 × 10^−8^
rs4760608	genotyped	A/C	0.349	0.966	0.057	−0.101 (0.025)	1.76 × 10^−4^
rs3782915	imputed	A/G	0.297	0.931	0.052	−0.302 (0.056)	4.22 × 10^−7^
rs740024	imputed	G/T	0.443	0.646	0.021	−0.171 (0.039)	9.34 × 10^−5^

Note: D’ and r^2^ denote linkage disequilibrium D’ and r^2^ values with the lead SNP rs2071358. MAF denotes minor allele frequency. β and SE denote effect size and related standard error.

## References

[b1] Moreno-Reyes R. . Kashin-Beck osteoarthropathy in rural Tibet in relation to selenium and iodine status. N Engl J Med 339, 1112–1120, doi: 10.1056/NEJM199810153391604 (1998). 9770558

[b2] Stone R. A medical mystery in middle China (vol, 324, pg 1378, 2009). Science. 325, 393–393 (2009). 10.1126/science.324_1378 19520932

[b3] Xiong G. Diagnostic, clinical and radiological characteristics of Kashin-Beck disease in Shaanxi Province, PR China. IntOrthop. 25, 147–150, doi: 10.1007/s002640100248 (2001). PMC3620652 11482528

[b4] Duan C. . Comparative Analysis of Gene Expression Profiles Between Primary Knee Osteoarthritis and an Osteoarthritis Endemic to Northwestern China, Kashin-Beck Disease. Arthritis Rheum-Us 62, 771–780, doi: 10.1002/art.27282 (2010). 20131229

[b5] Lu A. L. . Kashin-Beck disease and Sayiwak disease in China: Prevalence and a comparison of the clinical manifestations, familial aggregation, and heritability. Bone 48, 347–353, doi: 10.1016/j.bone.2010.09.015 (2011). 20854945

[b6] Xiong Y. M. . Association study between polymorphisms in selenoprotein genes and susceptibility to Kashin-Beck disease. Osteoarthr Cartilage 18, 817–824, doi: 10.1016/j.joca.2010.02.004 (2010). 20178852

[b7] Huang L. . Association study of polymorphisms in selenoprotein genes and Kashin-Beck disease and serum selenium/iodine concentration in a Tibetan population. PloS one 8, e71411, doi: 10.1371/journal.pone.0071411 (2013). 24058403 PMC3751926

[b8] Shi Y. . Genetic variants in the HLA-DRB1 gene are associated with Kashin-Beck disease in the Tibetan population. Arthritis Rheum 63, 3408–3416, doi: 10.1002/art.30526 (2011). 21739420

[b9] Zhang F. . Genome-wide copy number variation study and gene expression analysis identify ABI3BP as a susceptibility gene for Kashin-Beck disease. Human genetics 133, 793–799, doi: 10.1007/s00439-014-1418-4 (2014). 24442417

[b10] Du X. H. . SNP and mRNA expression for glutathione peroxidase 4 in Kashin-Beck disease. The British journal of nutrition 107, 164–169, doi: 10.1017/S0007114511002704 (2012). 21733339

[b11] Shi X., Zhang F., Lv A., Wen Y. & Guo X. COL9A1 gene polymorphism is associated with Kashin-Beck disease in a northwest Chinese Han population. PloS one 10, e0120365, doi: 10.1371/journal.pone.0120365 (2015). 25774918 PMC4361735

[b12] Du X. A. . Role of selenoprotein S (SEPS1) -105G>A polymorphisms and PI3K/Akt signaling pathway in Kashin-Beck disease. Osteoarthritis Cartilage 23, 210–216, doi: 10.1016/j.joca.2014.11.017 (2015). 25433273

[b13] Zhang F. . Genome-wide association study identifies ITPR2 as a susceptibility gene for Kashin-Beck disease in Han Chinese. Arthritis & rheumatology 67, 176–181, doi: 10.1002/art.38898 (2015). 25303641

[b14] Yang Z. . Whole-exome sequencing for the identification of susceptibility genes of Kashin-Beck disease. PloS one 9, e92298, doi: 10.1371/journal.pone.0092298 (2014). 24776925 PMC4002427

[b15] Zhao Q. M. . Association of TNF-alpha and Fas gene promoter polymorphism with the risk of Kashin-Beck disease in Northwest Chinese population. Clinical rheumatology 31, 1051–1057, doi: 10.1007/s10067-012-1975-7 (2012). 22431252

[b16] McAlinden A. Alternative splicing of type II procollagen: IIB or not IIB? Connective tissue research 55, 165–176, doi: 10.3109/03008207.2014.908860 (2014). 24669942 PMC4317353

[b17] Lui V. C., Ng L. J., Nicholls J., Tam P. P. & Cheah K. S. Tissue-specific and differential expression of alternatively spliced alpha 1 (II) collagen mRNAs in early human embryos. Developmental dynamics: an official publication of the American Association of Anatomists 203, 198–211, doi: 10.1002/aja.1002030208 (1995). 7655082

[b18] Oganesian A., Zhu Y. & Sandell, L. J. Type IIA procollagen amino propeptide is localized in human embryonic tissues. J HistochemCytochem 45, 1469–1480 (1997). 10.1177/002215549704501104 9358849

[b19] Sandell L. J., Morris N., Robbins J. R. & Goldring M. B. Alternatively spliced type II procollagen mRNAs define distinct populations of cells during vertebral development: differential expression of the amino-propeptide. The Journal of cell biology 114, 1307–1319 (1991). 1894696 10.1083/jcb.114.6.1307 PMC2289128

[b20] Li S. W. . Transgenic Mice with Targeted Inactivation of the Col2a1 Gene for Collagen-Ii Develop a Skeleton with Membranous and Periosteal Bone but No Endochondral Bone. Gene Dev 9, 2821–2830, doi: 10.1101/gad.9.22.2821 (1995). 7590256

[b21] Lewis R. . Disruption of the developmentally-regulated Col2a1 pre-mRNA alternative splicing switch in a transgenic knock-in mouse model. Matrix biology: journal of the International Society for Matrix Biology 31, 214–226, doi: 10.1016/j.matbio.2011.12.004 (2012). 22248926 PMC3295890

[b22] Kannu P., Bateman J. & Savarirayan R. Clinical phenotypes associated with type II collagen mutations. Journal of paediatrics and child health 48, E38–43, doi: 10.1111/j.1440-1754.2010.01979.x (2012). 21332586

[b23] Nishimura G. . The phenotypic spectrum of COL2A1 mutations. Human mutation **2** 6, 36–43, doi: 10.1002/humu.20179 (2005). 15895462

[b24] Gao Z. Q. . Altered aggrecan synthesis and collagen expression profiles in chondrocytes from patients with Kashin-Beck disease and osteoarthritis. The Journal of international medical research 40, 1325–1334 (2012). 22971484 10.1177/147323001204000411

[b25] Wang W., Guo X., Chen J., Xu P. & Lammi M. J. Morphology and phenotype expression of types I, II, III, and X collagen and MMP-13 of chondrocytes cultured from articular cartilage of Kashin-Beck Disease. J Rheumatol 35, 696–702, doi: 08/13/0313 (2008). 18322983

[b26] Guo X. . Recent advances in the research of an endemic osteochondropathy in China: Kashin-Beck disease. Osteoarthritis Cartilage 22, 1774–1783 (2014). 25106677 10.1016/j.joca.2014.07.023

[b27] Purcell S. . PLINK: A tool set for whole-genome association and population-based linkage analyses. Am J Hum Genet 81, 559–575, doi: 10.1086/519795 (2007). 17701901 PMC1950838

[b28] Hubisz M. J., Falush D., Stephens M. & Pritchard J. K. Inferring weak population structure with the assistance of sample group information. MolEcolResour 9, 1322–1332, doi: 10.1111/j.1755-0998.2009.02591.x (2009). PMC3518025 21564903

[b29] Price A. L. . Principal components analysis corrects for stratification in genome-wide association studies. Nat Genet 38, 904–909, doi: 10.1038/ng1847 (2006). 16862161

[b30] Marchini J., Howie B., Myers S., McVean G. & Donnelly P. A new multipoint method for genome-wide association studies by imputation of genotypes. Nat Genet 39, 906–913, doi: 10.1038/ng2088 (2007). 17572673

